# BRAF, NRAS, KIT, TERT, GNAQ/GNA11 Mutation Profile and Histomorphological Analysis of Anorectal Melanomas: A Clinicopathologic Study*

**DOI:** 10.5146/tjpath.2022.01576

**Published:** 2023-01-15

**Authors:** Orhun Cig Taskın, Sule Ozturk Sarı, Ismail Yılmaz, Ozge Hurdogan, Metin Keskin, Nesimi Buyukbabani, Mine Gulluoglu

**Affiliations:** Department of Pathology, Istanbul University, Istanbul Faculty of Medicine, Istanbul, Turkey; Sultan II. Abdulhamid Han Training and Research Hospital, University of Health Sciences, Istanbul, Turkey; Department of Surgery, Istanbul University, Istanbul Faculty of Medicine, Istanbul, Turkey

**Keywords:** Anorectal melanoma, BRAF, NRAS, KIT, TERT, GNA

## Abstract

***Objective:*** Primary anorectal melanomas (AMs) are uncommon neoplasms with aggressive behavior. Molecular profile and clinicopathologic features of AMs are still not well established. In this study, we aimed to investigate *BRAF, NRAS, KIT, TERT,* and *GNAQ/GNA11* mutation status and clinicopathologic features of AMs.

***Material and Method:*** All diagnostic slides of 15 AMs were reviewed. Histopathological and follow-up information were documented. Mutations in exon 15 of the *BRAF* gene; exons 2 and 3 of the *NRAS* gene; exons 9, 11, 13, 17, and 18 of the *KIT* gene; and exons 4 and 5 of the *GNAQ/GNA11* genes and mutations in the promoter region of the *TERT* gene (chr.5, 1,295,228C>T and 1,295,250C>T) were analyzed.

***Results:*** *BRAF*(V600E) and *KIT*(V555I and K642E) mutations were observed in one (7%) and two cases (14%), respectively. *NRAS, TERT* and *GNAQ/GNA11* mutations were not detected. The mean age was 65. Patients presented with rectal mass, rectal bleeding, pain, and weight loss. 73% of the lesions were macroscopically polypoid. The most common tumor cell type was epithelioid. Mean tumor thickness was 10.4 mm. One third of the cases lacked pigmentation. In situ melanoma was present in one third of the cases. Among 14 patients with follow-up data, 12 succumbed to disease. The mean overall survival was 36 months.

***Conclusion:*** AMs are uncommon tumors with dismal survival, usually occurring in the elderly in various gross and microscopic appearances. In terms of molecular profile, *BRAF* and *KIT* mutations are rarely detected. Profiling of larger cohorts is required to elucidate the pathogenesis and to identify potential molecular indicators that may contribute to the development of individualized targeted therapies.

## INTRODUCTION

Primary anorectal melanomas (AMs) are uncommon neoplasms that account for about 1% of anal canal tumors (1). Among mucosal melanomas, which constitute around 1% of all malignant melanomas (2), the anal canal is the second most common site of origin, following the head and neck (3). AMs are believed to arise from the melanocytes of the anal squamous epithelium and extend towards the anal canal (4,5); however, cases that originated from the rectal mucosa -without the involvement of the squamous epithelium- have also been reported (6,7).

Patients with AM usually present with rectal bleeding and pain. Tumors often mimic hemorrhoids, anal polyps or rectal carcinoma, forming large, dark-colored masses with expansile and nodular borders, with or without ulceration (8,9). Microscopically, tumors are often composed of sheets/fascicles of epithelioid or spindled malignant cells with vesicular chromatin and prominent nucleoli, with variable amounts of pigmentation (10). However, unusual presentations and rare histologic/cytologic patterns often challenge pathologists in the differential diagnosis of AMs, which includes carcinomas, sarcomas and even lymphomas (11,12). In challenging cases, a panel of immunohistochemical stains, including markers of melanocytic lineage is required to render the accurate diagnosis (13).

Similar to mucosal melanomas of other sites, AMs behave much worse than their cutaneous counterparts. Despite the use of various treatment regimens including extensive surgery, radiotherapy, chemotherapy, and targeted therapies, AMs have an aggressive clinical course with an overall 5-year survival rate of less than 25% (3,14). Additionally, AMs were associated with the poorest prognosis among mucosal melanomas in a large European cohort (15).

The recent progress in the molecular profiling of cutaneous melanomas has greatly contributed in our understanding of their pathogenesis, as well as their management with the use of targeted therapies and immunotherapy (16). However, mucosal melanomas tend to differ from their cutaneous counterparts in terms of molecular profiling; albeit showing a heterogeneous molecular profile, they have lower *BRAF *and* TERT*, and relatively higher *NRAS *and *KIT *mutation frequencies (17–27). Additionally,* GNAQ/GNA11* mutations, which have been reported in uveal melanomas and subjected to targeted therapies (28,29), also occur rarely in AMs (30), but not in other mucosal melanomas (31). However, molecular profiles of mucosal melanomas are still not well established due to their rareness. In addition to their molecular background, data concerning their clinicopathologic features are highly limited. Accordingly, widely accepted treatment protocols do not exist. In this study, as an extension to our previous work on head and neck mucosal melanomas (31), we aimed to investigate the *BRAF*, *NRAS*, *KIT*, *TERT* and *GNAQ/GNA11* mutation status of 15 AMs, as well as their clinicopathologic features.

## MATERIAL and METHODS

### Case Selection, Clinical and Pathological Data Collection

The digital database of the pathology department (Istanbul Faculty of Medicine, Istanbul University, Istanbul, Turkey) was searched for cases diagnosed as AM between the years 2000 and 2019, including both in-house material and outside consultations. Data on clinical history and physical/radiologic examination were reviewed for all retrieved cases in order to exclude previous history of cutaneous melanoma and/or the possibility of metastasis. Cases with a suspicion of secondary melanoma were not included.

Diagnostic slides and paraffin blocks were retrieved from the archives. All slides (hematoxylin and eosin and/or immunohistochemically stained) were reviewed. Tumor cell types were classified under four categories (epithelioid, spindle, pleomorphic, and lymphoma-like), as mentioned in the literature (9,12,32,33), and combined morphology was assessed when appropriate. Histopathological information regarding tumor thickness (Breslow), presence of perineural and lymphovascular invasion, pigmentation, ulceration, necrosis, tumor infiltrating lymphocytes, mitotic count (per high power field), and margin status (when applicable) were also documented. Follow-up information was obtained from the clinical files or national database.

### Mutation Analysis

Tumor targets (>90% viable tumor) were manually microdissected from 10-mm thick unstained histologic sections for enrichment of tumor cellularity. Deparaffinization of tissue sections was performed. Then, DNA was isolated by using the QIAamp DNA FFPE Tissue Kit (50) (catalog #: 56404) (QIAGEN, Hilden, Germany). DNA concentrations of the samples were assessed spectrophotometrically using a Nanodrop 1000 spectrophotometer (ThermoScientific, USA).

Mutations in exon 15 of *BRAF* gene; exons 2 and 3 of the *NRAS* gene; exons 9, 11, 13, 17, and 18 of the *KIT* gene; and exons 4 and 5 of the *GNAQ* and *GNA11* genes (well-known hotspot regions for oncogenic mutations) and mutations in the promoter region of the *TERT *gene (chr5, 1,295,228C>T and 1,295,250C>T) were analyzed by validated previously described polymerase chain reaction (PCR)-based direct Sanger sequencing (analytical sensitivity 25%) by using 200 ng of each tumor DNA (31).

### Additional Information

The Helsinki principles were respected in this study and patients’ data confidentiality was ensured according to their guidelines. This study was approved by the institutional review board.

## RESULTS

### Clinical Features

The study was conducted with 15 cases of 15 patients (8 males and 7 females) with a mean age of 65 years (range: 30 - 86 years). The specimens consisted of 8 local excisions, 3 abdominoperineal resections, 1 polypectomy and 3 incisional biopsies. By definition, all tumors originated from the anal canal. Among patients with available information (n=9), presenting symptoms were described as rectal mass, rectal bleeding, pain and weight loss.

### Histopathology

**Figure 1:**
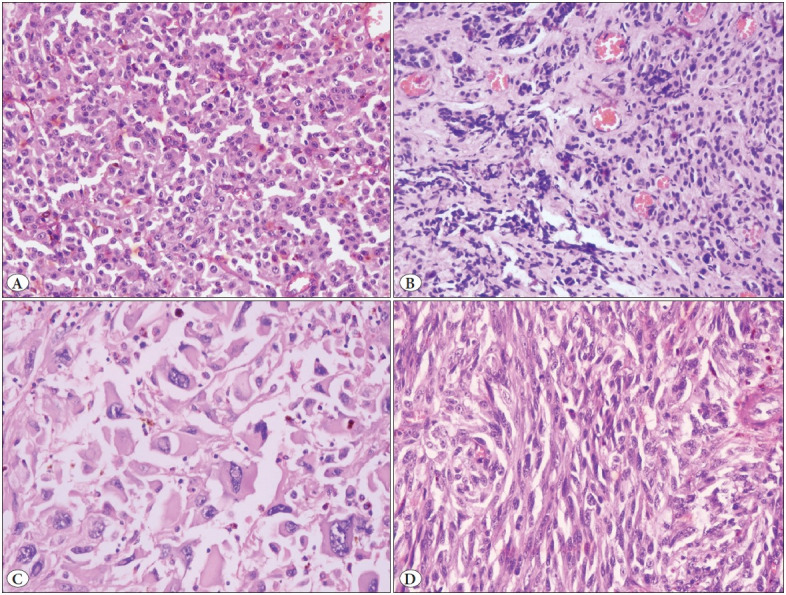
Neoplastic cells in anorectal melanoma demonstrate various morphologic appearances: **A)** Epithelioid melanoma cells with roundish nuclei and wide eosinophilic cytoplasm, **B)** Lymphoma-like small neoplastic cells, admixed in a fibrous stroma, showing crush artifact, **C)** Pleomorphic melanoma cells with huge, bizarre nuclei and prominent cytoplasm, **D)** Spindle cells showing elongated nuclei and sparse cytoplasm (**A-D**: Hematoxylin&Eosin, x400).

**Figure 2:**
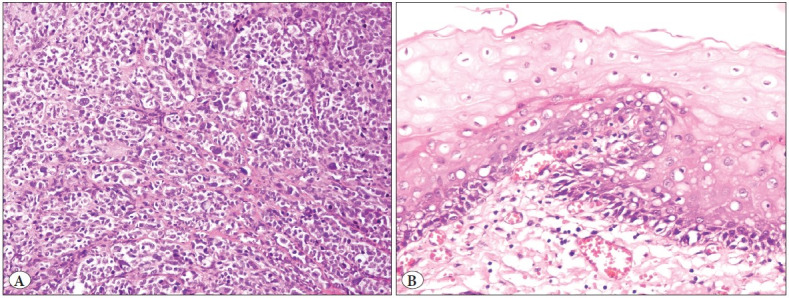
**A)** Anorectal melanoma cells, which lack melanin pigment (Hematoxylin&Eosin, x200), **B)** Melanoma in situ, atypical melanocytes showing continuous growth at the basal layer (Hematoxylin&Eosin, x400).

Diagnostic slides of the fifteen cases were systematically reviewed. Tumors were polypoid in 73%. The cell type was epithelioid and spindle in 33%, epithelioid and lymphoma-like in 27%, spindle in 13%, epithelioid and pleomorphic in 13%, spindle and lymphoma-like in 7%, and lymphoma-like in 7% of cases (Figure 1). Mean tumor thickness in 12 cases was 10.4 mm (range: 1.1-22 mm). Tumor thickness could not be measured in 2 incisional biopsies and 1 polypectomy due to poor orientation. Majority of cases (67%) showed pigmentation, whereas 33% were amelanotic (Figure 2). Ulceration was seen in 80% of cases. Mean mitotic count was 4.9 per 10 high power fields (range 0-10). In situ melanoma was detected in 33% of the cases (Figure 2). Intratumoral lymphocytes were prominent in 53%. Metastasis in lymph nodes was observed in 3 abdominoperineal resections.

### Immunohistochemistry

Among 15 cases, 10 were subjected to immunohistochemical analysis. Five cases that did not require immunohistochemical analysis harbored in situ melanoma component and/or prominent pigmentation.

Among melanocytic markers, S-100 and HMB-45 were positive in all cases (100%; n=9 and 7; respectively). Melan-A was positive in 6/7 (86%) cases. Epithelial markers (Pan-cytokeratin, epithelial membrane antigen, and carcinoembryonic antigen), neuroendocrine markers (chromogranin, synaptophysin), muscle markers (desmin, smooth muscle actin) were all negative when performed, along with leukocyte common antigen, CD30 and CD34. CD117 was positive in 2 of 3 cases performed.

### Mutation Analysis

A total of 3 cases (20%) were found to harbor mutations. *BRAF* (V600E) and *KIT *(V555I and K642E) mutations were observed in one (7%) and two cases (14%), respectively. *NRAS*, *TERT,* and *GNAQ/GNA11* mutations were not observed.

### Follow-Up and Survival Information

Among 14 patients with available information, 12 died. The mean overall survival was 36 months (range: 0-112 months). The histopathological, clinical, and mutational findings and follow-up information are summarized in Table 1.

**Table 1:** Clinicopathologic features of the study group.

**Case No**	**Age**	**Sex**	**Operation Type**	**Thickness (mm)**	**Ulceration**	**Mitosis (n/HPF)**	**In situ melanoma**	**Intratumoral lymphocytes**	**Vascular invasion**	**Perineural invasion**	**Necrosis**	**Pigment**	**Cell type**	**Surgical margins**	**Patient status**	**Overall survival (months)**	**Mutation**
1	82	M	Local excision	10.0	Present	3	Absent	Present	Absent	Absent	Absent	Present	Epithelioid & spindle	N/A	Dead	70	*BRAF* (1799T>A, V600E)
2	63	M	Local excision	22.0	Present	3	Present	Absent	Absent	Absent	Absent	Present	Epithelioid & pleomorphic	Positive	Dead	4	None
3	86	F	Incisional biopsy	N/A	Present	4	Absent	Absent	Absent	Absent	Absent	Present	Epithelioid & spindle	N/A	Dead	0	None
4	68	M	Incisional biopsy	N/A	Present	0	Present	Absent	Absent	Absent	Absent	Absent	Epithelioid & lymphoma-like	N/A	Lost to follow-up	Lost to follow-up	None
5	75	F	Local excision	9.4	Present	6	Present	Present	Present	Present	Absent	Absent	Epithelioid & pleomorphic	Positive	Dead	5	None
6	58	F	Local excision	7.7	Present	6	Present	Present	Absent	Absent	Absent	Present	Epithelioid & spindle	N/A	Alive	85	None
7	72	F	Local excision	11.0	Present	9	Absent	Absent	Absent	Absent	Absent	Present	Epithelioid & lymphoma-like	Negative	Dead	28	*KIT* Exon 11 (1663G>A, V555I)
8	71	F	Abdominoperineal resection	13.0	Present	2	Absent	Absent	Present	Absent	Absent	Absent	Spindle	Negative	Alive	112	KIT Exon 13 (1924A>G, K642E)
9	69	M	Incisional biopsy	9.0	Absent	9	Absent	Present	Absent	Absent	Absent	Present	Spindle	N/A	Dead	52	None
10	52	M	Polypectomy	N/A	Absent	0	Absent	Absent	Absent	Absent	Absent	Present	Lymphoma-like	N/A	Dead	21	None
11	68	F	Local excision	1.1	Present	8	Absent	Absent	Absent	Absent	Absent	Absent	Epithelioid & lymphoma-like	Positive	Dead	87	None
12	46	M	Local excision	14.0	Present	6	Present	Absent	Absent	Absent	Absent	Present	Epithelioid & spindle	Positive	Dead	21	None
13	65	M	Abdominoperineal resection	12.0	Absent	6	Absent	Absent	Present	Absent	Absent	Present	Epithelioid & lymphoma-like	Negative	Dead	7	None
14	30	M	Abdominoperineal resection	11.0	Present	10	Absent	Present	Absent	Present	Positive	Present	Spindle & lymphoma-like	Positive	Dead	11	None
15	65	F	Local excision	4.5	Present	2	Absent	Absent	Absent	Absent	Absent	Absent	Epithelioid & spindle	N/A	Dead	6	None

**N/A:** Not applicable.

## DISCUSSION

Mutational profile of mucosal melanomas is known to differ from their cutaneous counterparts, suggesting a different pathway in the pathogenesis: They harbor lower *BRAF* and *TERT*, and relatively higher *NRAS* and *KIT* mutation frequencies (17–27). The absence of UV damage is often mentioned to be associated with this disparity. Furthermore, regarding the site of origin, differences also exist in the same subgroup: in an earlier study, we concluded that *NRAS* and *TERT* promoter mutation rates were significantly higher in sinonasal than in oral mucosal melanomas of the head and neck (31). In the current literature, data on AMs’ molecular profile is mostly merged with cutaneous and/or mucosal melanomas, primarily due to their rareness (34–39). In studies with relatively large cohorts of AMs, *KIT* mutations were most commonly encountered, followed by mutations in *NRAS*. The newly introduced *NF1 *gene also has an important role in the oncogenesis. *BRAF* mutations were also observed with different frequencies, most likely due to small sample sizes or populational differences of the cohorts (40–44). In addition, one study showed around 2% *GNAQ* and 6% *GNA11* mutations (39). In our study group, *BRAF* and *KIT *mutations were found in 7% and 14%, respectively. *NRAS,* *TERT,* and *GNAQ/GNA11* mutations were absent. Together, these supported the low mutation burden of AMs, as stated in the literature (45). In a large cohort of mucosal melanomas, 3% of AMs showed *BRAF*, 10% showed *NRAS*, and 19% showed *KIT* mutations. This study analyzed a subset of cases by Sanger sequencing, and others by next-generation sequencing (NGS); and proposed *NRAS* mutation as a predictor of worse survival, independent of stage in all mucosal melanomas (46). Those being mentioned, as a limitation of this study, we had a limited number of cases, impeding a correlation analysis between mutational status and prognostic data. We also did not have access to NGS techniques. Therefore we were unable to perform a comprehensive genomic analysis including *NF1*, which was recently integrated in the molecular classification of AMs.

Our findings verified that AMs are highly rare and aggressive neoplasms that generally occur in elder patients, with a mean age of 65 years in the present study. In one study, older age (>70 years) was found to be an independent poor prognostic factor (10). Although our data did not reveal any significant sex predilection, geographic and populational differences in the relative frequency between two genders have been reported (10,47).

Clinically, recognizing AMs can be challenging for physicians. The symptomatology may include non-specific rectal bleeding and pain, as well as weight loss in metastatic disease (48). Endoscopically, tumors can present with various appearances. Polypoid masses are frequently encountered, similar to 73% of our cases. Anal prolapse, and luminal or submucosal masses with or without ulceration or pigmentation can also be seen (48). This may cause misdiagnosis of AM as hemorrhoids, perianal abscess, anal polyps or other malignancies (49).

The presence of melanin pigmentation can help render the accurate diagnosis. However, it is not always present, with some studies reporting 37% of their cases as amelanotic (12,32). In addition, in situ melanoma component, or junctional melanocytic activity, which are characteristic in cutaneous melanomas, have been reported in up to 75% of AMs (12,32,49). However, this feature may be missing due to the absence of adjacent mucosa in incisional biopsies that consist entirely of tumor, and also due to ulceration and fragmentation in excisional biopsies. In our study, a third of the tumors were amelanotic and a third had an in situ component.

Microscopically, epithelioid, spindled, pleomorphic, and lymphoma-like tumor cells may co-exist, with epithelioid being the most frequent with combination of the others (32,40), similar to the present study. Therefore, AMs can mimic a large spectrum of malignancies, making the use of immunohistochemistry crucial in differential diagnosis. Additionally, lack of in situ component and/or lack of pigmentation, also complicate the diagnostic puzzle. At this point, an immunohistochemical panel of commonly used melanocytic markers, S-100 protein/SOX10, Melan-A, HMB-45, can be helpful. Moreover, additional markers may be required to rule out other entities including primary or metastatic carcinomas, neuroendocrine neoplasms, sarcomas, lymphomas, and gastrointestinal stromal tumors. Among those, the use of CD117 requires careful interpretation due to its frequent positivity in AMs (up to 75% in the literature), which can lead to a misdiagnosis of rectal gastrointestinal stromal tumor, if not performed along with other melanocytic markers (12,33). Additionally, CD117 immunohistochemistry is known not to correlate with KIT status and therefore should not be used with mutation screening purposes (44).

In terms of pathological staging and prognosis, specific guidelines for reporting AMs do not exist. They are usually reported according to the American Joint Commission on Cancer (AJCC) guidelines for cutaneous melanoma (50), which depends mostly on tumor thickness, causing several issues in the daily practice. In the vast majority of cases on reported series (10,40,45,51–53) including ours (10.4 mm), the average tumor thickness was much thicker than the 4 mm threshold used for staging T4 cutaneous melanomas. This threshold inevitably categorizes the bulk of cases as T4, thus diminishing the prognostic stratification of the T classification. Several attempts have been made in order to sharpen the prognostic accuracy, including the implementation of different thickness cut-offs (51), subclassification depending on the localization (52) and metastatic status (40). Among other histopathologic prognostic factors, presence of metastasis, lymphovascular and perineural invasion, invasion of muscularis propria/anal sphincter were also reported (10,40,51). Mitotic rate is a very strong prognostic factor in cutaneous melanomas (54). Although high mitotic rates are frequently encountered similar to our study, their correlation with the clinical outcome is not well established in AMs (12,33). Nevertheless, studies on larger cohorts are needed in order to define the relationship between the distinct histopathologic parameters and prognosis.

In terms of treatment, optimal algorithms are lacking and satisfactory results are yet to be achieved (55). The primary choice of treatment is complete surgical removal of the tumor (8). Advantages of local approaches (mucosal resection or local excision) over extensive surgery (abdominoperineal resection) have long been discussed; however, literature data lack proof to recommend one modality over the other (56–58). Moreover, adjuvant or neoadjuvant therapies do not seem to make significant difference on the clinical outcome (55). The results of recently implemented immunotherapy is yet to be proven (59). Since our data involved limited information on adjuvant treatment, we were unable to draw any conclusions on this subject.

In conclusion, AMs are uncommon tumors with aggressive behavior and poor survival. They usually occur in the elderly and present in various gross and microscopic appearances, thus involving a wide spectrum of differential diagnoses. For accurate diagnosis, the melanocytic lineage should be demonstrated with immunohistochemistry, especially in the absence of conventional morphological clues such as pigmentation and/or in situ component. In terms of molecular profile, *BRAF *and *KIT* mutations rarely occur. Profiling of larger cohorts is required to elucidate the pathogenesis and to identify potential molecular indicators that may contribute in the development of individualized targeted therapies.

## Conflict of Interest and Funding Statement

Authors have no conflicts of interest to declare. All authors have read and contributed to the final manuscript and confirm that this is an original work that has not been previously published, nor has it been submitted to another journal for simultaneous review. This study is supported by the Scientific Research Project Fund of Istanbul University (Project number: 51524). This study was partially presented in 28th Congress of the European Society of Pathology, 25-29 September 2016, Cologne, Germany.

## References

[ref-1] Belbaraka Rhizlane, Elharroudi Tijani, Ismaili Nabil, Fetohi Mohammed, Tijami Fouad, Jalil Abdelouahed, Errihani Hassan (2012). Management of anorectal melanoma: report of 17 cases and literature review. J Gastrointest Cancer.

[ref-2] Chen Haiyan, Cai Yibo, Liu Yue, He Jinjie, Hu Yeting, Xiao Qian, Hu Wangxiong, Ding Kefeng (2016). Incidence, Surgical Treatment, and Prognosis of Anorectal Melanoma From 1973 to 2011: A Population-Based SEER Analysis. Medicine (Baltimore).

[ref-3] Chang A. E., Karnell L. H., Menck H. R. (1998). The National Cancer Data Base report on cutaneous and noncutaneous melanoma: a summary of 84,836 cases from the past decade. The American College of Surgeons Commission on Cancer and the American Cancer Society. Cancer.

[ref-4] Ackermann D. M., Polk H. C., Schrodt G. R. (1985). Desmoplastic melanoma of the anus. Hum Pathol.

[ref-5] Morson B. C., Volkstädt H. (1963). Malignant melanoma of the anal canal. J Clin Pathol.

[ref-6] Nicholson A. G., Cox P. M., Marks C. G., Cook M. G. (1993). Primary malignant melanoma of the rectum. Histopathology.

[ref-7] Werdin C., Limas C., Knodell R. G. (1988). Primary malignant melanoma of the rectum. Evidence for origination from rectal mucosal melanocytes. Cancer.

[ref-8] Malaguarnera Giulia, Madeddu Roberto, Catania Vito Emanuele, Bertino Gaetano, Morelli Luca, Perrotta Rosario Emanuele, Drago Filippo, Malaguarnera Michele, Latteri Saverio (2018). Anorectal mucosal melanoma. Oncotarget.

[ref-9] Cruz Geraldo Magela Gomes Da, Filho José De Souza Andrade, Patrus Gil, Leite Sinara Mônica De Oliveira, Da Silva Ilson Geraldo, Teixeira Ricardo Guimarães, Braga Áurea Cassia Gualberto, Ferreira Renata Magali Ribeiro Silluzio (2014). Anorectal melanoma – histopathological and immunohistochemical features and treatment. J Coloproctology.

[ref-10] Ren Min, Lu Yawen, Lv Jiaojie, Shen Xuxia, Kong Jincheng, Dai Bo, Kong Yunyi (2018). Prognostic factors in primary anorectal melanoma: a clinicopathological study of 60 cases in China. Hum Pathol.

[ref-11] Nakhleh R. E., Wick M. R., Rocamora A., Swanson P. E., Dehner L. P. (1990). Morphologic diversity in malignant melanomas. Am J Clin Pathol.

[ref-12] Tariq Muhammad Usman, Ud Din Nasir, Ud Din Nausheen Feroz, Fatima Saira, Ahmad Zubair (2014). Malignant melanoma of anorectal region: a clinicopathologic study of 61 cases. Ann Diagn Pathol.

[ref-13] Prieto Victor G., Shea Christopher R. (2011). Immunohistochemistry of melanocytic proliferations. Arch Pathol Lab Med.

[ref-14] Singer Marc, Mutch Matthew G. (2006). Anal melanoma. Clin Colon Rectal Surg.

[ref-15] Heppt Markus V., Roesch Alexander, Weide Benjamin, Gutzmer Ralf, Meier Friedegund, Loquai Carmen, Kähler Katharina C., Gesierich Anja, Meissner Markus, Bubnoff Dagmar, Göppner Daniela, Schlaak Max, Pföhler Claudia, Utikal Jochen, Heinzerling Lucie, Cosgarea Ioana, Engel Jutta, Eckel Renate, Martens Alexander, Mirlach Laura, Satzger Imke, Schubert-Fritschle Gabriele, Tietze Julia K., Berking Carola (2017). Prognostic factors and treatment outcomes in 444 patients with mucosal melanoma. Eur J Cancer.

[ref-16] Melis C., Rogiers A., Bechter O., Oord Joost J. (2017). Molecular genetic and immunotherapeutic targets in metastatic melanoma. Virchows Arch.

[ref-17] Tacastacas Joselin D., Bray Julie, Cohen Yoon K., Arbesman Joshua, Kim Julian, Koon Henry B., Honda Kord, Cooper Kevin D., Gerstenblith Meg R. (2014). Update on primary mucosal melanoma. J Am Acad Dermatol.

[ref-18] Guo Jun, Si Lu, Kong Yan, Flaherty Keith T., Xu Xiaowei, Zhu Yanyan, Corless Christopher L., Li Li, Li Haifu, Sheng Xinan, Cui Chuanliang, Chi Zhihong, Li Siming, Han Mei, Mao Lili, Lin Xuede, Du Nan, Zhang Xiaoshi, Li Junling, Wang Baocheng, Qin Shukui (2011). Phase II, open-label, single-arm trial of imatinib mesylate in patients with metastatic melanoma harboring c-Kit mutation or amplification. J Clin Oncol.

[ref-19] Miao Yuwen, Wang Runxiang, Ju Houyu, Ren Guoxin, Guo Wei, Lyu Jiong (2015). TERT promoter mutation is absent in oral mucosal melanoma. Oral Oncol.

[ref-20] Hodi F. Stephen, Corless Christopher L., Giobbie-Hurder Anita, Fletcher Jonathan A., Zhu Meijun, Marino-Enriquez Adrian, Friedlander Philip, Gonzalez Rene, Weber Jeffrey S., Gajewski Thomas F., O'Day Steven J., Kim Kevin B., Lawrence Donald, Flaherty Keith T., Luke Jason J., Collichio Frances A., Ernstoff Marc S., Heinrich Michael C., Beadling Carol, Zukotynski Katherine A., Yap Jeffrey T., Abbeele Annick D., Demetri George D., Fisher David E. (2013). Imatinib for melanomas harboring mutationally activated or amplified KIT arising on mucosal, acral, and chronically sun-damaged skin. J Clin Oncol.

[ref-21] Carvajal Richard D., Antonescu Cristina R., Wolchok Jedd D., Chapman Paul B., Roman Ruth-Ann, Teitcher Jerrold, Panageas Katherine S., Busam Klaus J., Chmielowski Bartosz, Lutzky Jose, Pavlick Anna C., Fusco Anne, Cane Lauren, Takebe Naoko, Vemula Swapna, Bouvier Nancy, Bastian Boris C., Schwartz Gary K. (2011). KIT as a therapeutic target in metastatic melanoma. JAMA.

[ref-22] Beadling Carol, Jacobson-Dunlop Erick, Hodi F. Stephen, Le Claudia, Warrick Andrea, Patterson Janice, Town Ajia, Harlow Amy, Cruz Frank, Azar Sharl, Rubin Brian P., Muller Susan, West Rob, Heinrich Michael C., Corless Christopher L. (2008). KIT gene mutations and copy number in melanoma subtypes. Clin Cancer Res.

[ref-23] Curtin John A., Busam Klaus, Pinkel Daniel, Bastian Boris C. (2006). Somatic activation of KIT in distinct subtypes of melanoma. J Clin Oncol.

[ref-24] Minor David R., Kashani-Sabet Mohammed, Garrido Maria, O'Day Steven J., Hamid Omid, Bastian Boris C. (2012). Sunitinib therapy for melanoma patients with KIT mutations. Clin Cancer Res.

[ref-25] Griewank Klaus G., Murali Rajmohan, Puig-Butille Joan Anton, Schilling Bastian, Livingstone Elisabeth, Potrony Miriam, Carrera Cristina, Schimming Tobias, Möller Inga, Schwamborn Marion, Sucker Antje, Hillen Uwe, Badenas Celia, Malvehy Josep, Zimmer Lisa, Scherag André, Puig Susana, Schadendorf Dirk (2014). TERT Promoter Mutation Status as an Independent Prognostic Factor in Cutaneous Melanoma. J Natl Cancer Inst.

[ref-26] Egberts Friederike, Krüger Sandra, Behrens Hans M., Bergner Inka, Papaspyrou Giorgios, Werner Jochen A., Alkatout Ibrahim, Haag Jochen, Hauschild Axel, Röcken Christoph (2014). Melanomas of unknown primary frequently harbor TERT-promoter mutations. Melanoma Res.

[ref-27] Jangard Mattias, Zebary Abdlsattar, Ragnarsson-Olding Boel, Hansson Johan (2015). TERT promoter mutations in sinonasal malignant melanoma: a study of 49 cases. Melanoma Res.

[ref-28] Van Raamsdonk Catherine D., Bezrookove Vladimir, Green Gary, Bauer Jürgen, Gaugler Lona, O'Brien Joan M., Simpson Elizabeth M., Barsh Gregory S., Bastian Boris C. (2009). Frequent somatic mutations of GNAQ in uveal melanoma and blue naevi. Nature.

[ref-29] Chen X., Wu Q., Tan L., Porter D., Jager M. J., Emery C., Bastian B. C. (2014). Combined PKC and MEK inhibition in uveal melanoma with GNAQ and GNA11 mutations. Oncogene.

[ref-30] Kim Chung-Young, Kim Dae Won, Kim Kevin, Curry Jonathan, Torres-Cabala Carlos, Patel Sapna (2014). GNAQ mutation in a patient with metastatic mucosal melanoma. BMC Cancer.

[ref-31] Öztürk Sari Şule, Yilmaz İsmaİl, Taşkin Orhun Çiğ, Narli Gİzem, Şen Fatma, Çomoğlu Şenol, Firat Pinar, Bİlgİç Bİlge, Yilmazbayhan Dİlek, Özlük Yasemİn, Büyükbabanİ Nesİmİ (2017). BRAF, NRAS, KIT, TERT, GNAQ/GNA11 mutation profile analysis of head and neck mucosal melanomas: a study of 42 cases. Pathology.

[ref-32] Charifa Ahmad, Zhang Xuchen (2018). Morphologic and Immunohistochemical Characteristics of Anorectal Melanoma. Int J Surg Pathol.

[ref-33] Chute Deborah J., Cousar John B., Mills Stacey E. (2006). Anorectal malignant melanoma: morphologic and immunohistochemical features. Am J Clin Pathol.

[ref-34] Omholt Katarina, Grafström Eva, Kanter-Lewensohn Lena, Hansson Johan, Ragnarsson-Olding Boel K. (2011). KIT pathway alterations in mucosal melanomas of the vulva and other sites. Clin Cancer Res.

[ref-35] Quek Camelia, Rawson Robert V., Ferguson Peter M., Shang Ping, Silva Ines, Saw Robyn P. M., Shannon Kerwin, Thompson John F., Hayward Nicholas K., Long Georgina V., Mann Graham J., Scolyer Richard A., Wilmott James S. (2019). Recurrent hotspot SF3B1 mutations at codon 625 in vulvovaginal mucosal melanoma identified in a study of 27 Australian mucosal melanomas. Oncotarget.

[ref-36] Schaefer Tim, Satzger Imke, Gutzmer Ralf (2017). Clinics, prognosis and new therapeutic options in patients with mucosal melanoma: A retrospective analysis of 75 patients. Medicine (Baltimore).

[ref-37] Edwards R. H., Ward M. R., Wu H., Medina C. A., Brose M. S., Volpe P., Nussen-Lee S., Haupt H. M., Martin A. M., Herlyn M., Lessin S. R., Weber B. L. (2004). Absence of BRAF mutations in UV-protected mucosal melanomas. J Med Genet.

[ref-38] Newell Felicity, Kong Yan, Wilmott James S., Johansson Peter A., Ferguson Peter M., Cui Chuanliang, Li Zhongwu, Kazakoff Stephen H., Burke Hazel, Dodds Tristan J., Patch Ann-Marie, Nones Katia, Tembe Varsha, Shang Ping, Weyden Louise, Wong Kim, Holmes Oliver, Lo Serigne, Leonard Conrad, Wood Scott, Xu Qinying, Rawson Robert V., Mukhopadhyay Pamela, Dummer Reinhard, Levesque Mitchell P., Jönsson Göran, Wang Xuan, Yeh Iwei, Wu Hong, Joseph Nancy, Bastian Boris C., Long Georgina V., Spillane Andrew J., Shannon Kerwin F., Thompson John F., Saw Robyn P. M., Adams David J., Si Lu, Pearson John V., Hayward Nicholas K., Waddell Nicola, Mann Graham J., Guo Jun, Scolyer Richard A. (2019). Whole-genome landscape of mucosal melanoma reveals diverse drivers and therapeutic targets. Nat Commun.

[ref-39] Sheng Xinan, Kong Yan, Li Yiqian, Zhang Qiannan, Si Lu, Cui Chuanliang, Chi Zhihong, Tang Bixia, Mao Lili, Lian Bin, Wang Xuan, Yan Xieqiao, Li Siming, Dai Jie, Guo Jun (2016). GNAQ and GNA11 mutations occur in 9.5% of mucosal melanoma and are associated with poor prognosis. Eur J Cancer.

[ref-40] Nagarajan Priyadharsini, Piao Jin, Ning Jing, Noordenbos Laura E., Curry Jonathan L., Torres-Cabala Carlos A., Diwan A. Hafeez, Ivan Doina, Aung Phyu P., Ross Merrick I., Royal Richard E., Wargo Jennifer A., Wang Wei-Lien, Samdani Rashmi, Lazar Alexander J., Rashid Asif, Davies Michael A., Prieto Victor G., Gershenwald Jeffrey E., Tetzlaff Michael T. (2020). Prognostic model for patient survival in primary anorectal mucosal melanoma: stage at presentation determines relevance of histopathologic features. Mod Pathol.

[ref-41] Yang Hui Min, Hsiao Susan J., Schaeffer David F., Lai Chi, Remotti Helen E., Horst David, Mansukhani Mahesh M., Horst Basil A. (2017). Identification of recurrent mutational events in anorectal melanoma. Mod Pathol.

[ref-42] Ni Shujuan, Huang Dan, Chen Xiaochen, Huang Jiaying, Kong Yunyi, Xu Ye, Du Xiang, Sheng Weiqi (2012). c-kit gene mutation and CD117 expression in human anorectal melanomas. Hum Pathol.

[ref-43] Hintzsche Jennifer D., Gorden Nicholas T., Amato Carol M., Kim Jihye, Wuensch Kelsey E., Robinson Steven E., Applegate Allison J., Couts Kasey L., Medina Theresa M., Wells Keith R., Wisell Joshua A., McCarter Martin D., Box Neil F., Shellman Yiqun G., Gonzalez Rene C., Lewis Karl D., Tentler John J., Tan Aik Choon, Robinson William A. (2017). Whole-exome sequencing identifies recurrent SF3B1 R625 mutation and comutation of NF1 and KIT in mucosal melanoma. Melanoma Res.

[ref-44] Santi Raffaella, Simi Lisa, Fucci Rossella, Paglierani Milena, Pepi Monica, Pinzani Pamela, Merelli Barbara, Santucci Marco, Botti Gerardo, Urso Carmelo, Massi Daniela (2015). KIT genetic alterations in anorectal melanomas. J Clin Pathol.

[ref-45] Dodds Tristan J., Wilmott James S., Jackett Louise A., Lo Serigne N., Long Georgina V., Thompson John F., Scolyer Richard A. (2019). Primary anorectal melanoma: clinical, immunohistology and DNA analysis of 43 cases. Pathology.

[ref-46] Wróblewska Joanna P., Dias-Santagata Dora, Ustaszewski Adam, Wu Cheng-Lin, Fujimoto Masakazu, Selim M. Angelica, Biernat Wojciech, Ryś Janusz, Marszalek Andrzej, Hoang Mai P. (2021). Prognostic Roles of BRAF, KIT, NRAS, IGF2R and SF3B1 Mutations in Mucosal Melanomas. Cells.

[ref-47] Callahan Adrienne, Anderson William F., Patel Sital, Barnholtz-Sloan Jill S., Bordeaux Jeremy S., Tucker Margaret A., Gerstenblith Meg R. (2016). Epidemiology of Anorectal Melanoma in the United States: 1992 to 2011. Dermatol Surg.

[ref-48] La Selva Danielle, Kozarek Richard A., Dorer Russell K., Rocha Flavio G., Gluck Michael (2020). Primary and metastatic melanoma of the GI tract: clinical presentation, endoscopic findings, and patient outcomes. Surg Endosc.

[ref-49] Cooper P. H., Mills S. E., Allen M. S. (1982). Malignant melanoma of the anus: report of 12 patients and analysis of 255 additional cases. Dis Colon Rectum.

[ref-50] Amin Mahul B., Edge Stephen B., Greene Frederick L., Byrd David R., Brookland Robert K., Washington Mary Kay, Gershenwald Jeffrey E., Compton Carolyn C., Hess Kenneth R., Sullivan Daniel C., Jessup J. Milburn, Brierley James D., Gaspar Lauri E., Schilsky Richard L., Balch Charles M., Winchester David P., Asare Elliot A., Madera Martin, Gress Donna M., Meyer Laura R. (2017). AJCC Cancer Staging Manual.

[ref-51] Yeh Jen Jen, Shia Jinru, Hwu Wen Jen, Busam Klaus J., Paty Philip B., Guillem Jose G., Coit Daniel G., Wong W. Douglas, Weiser Martin R. (2006). The role of abdominoperineal resection as surgical therapy for anorectal melanoma. Ann Surg.

[ref-52] Bello Danielle M., Smyth Elizabeth, Perez Daniel, Khan Shaheer, Temple Larissa K., Ariyan Charlotte E., Weiser Martin R., Carvajal Richard D. (2013). Anal versus rectal melanoma: does site of origin predict outcome?. Dis Colon Rectum.

[ref-53] Ben-Izhak O., Levy R., Weill S., Groisman G., Cohen H., Stajerman S., Misselevich I., Nitecky S., Eidelman S., Kerner H. (1997). Anorectal malignant melanoma. A clinicopathologic study, including immunohistochemistry and DNA flow cytometry. Cancer.

[ref-54] Piñero-Madrona A., Ruiz-Merino G., Cerezuela Fuentes P., Martínez-Barba E., Rodríguez-López J. N., Cabezas-Herrera J. (2019). Mitotic rate as an important prognostic factor in cutaneous malignant melanoma. Clin Transl Oncol.

[ref-55] Kirchoff Daniel D., Deutsch Gary B., Foshag Leland J., Lee Ji Hey, Sim Myung-Shin, Faries Mark B. (2016). Evolving Therapeutic Strategies in Mucosal Melanoma Have Not Improved Survival Over Five Decades. Am Surg.

[ref-56] Kelly Patrick, Zagars Gunar K., Cormier Jancie N., Ross Merrick I., Guadagnolo B. Ashleigh (2011). Sphincter-sparing local excision and hypofractionated radiation therapy for anorectal melanoma: a 20-year experience. Cancer.

[ref-57] Ross M., Pezzi C., Pezzi T., Meurer D., Hickey R., Balch C. (1990). Patterns of failure in anorectal melanoma. A guide to surgical therapy. Arch Surg.

[ref-58] Che Xu, Zhao Dong-Bing, Wu Yong-Kai, Wang Cheng-Feng, Cai Jian-Qiang, Shao Yong-Fu, Zhao Ping (2011). Anorectal malignant melanomas: retrospective experience with surgical management. World J Gastroenterol.

[ref-59] Taylor James P., Stem Miloslawa, Yu David, Chen Sophia Y., Fang Sandy H., Gearhart Susan L., Safar Bashar, Efron Jonathan E. (2019). Treatment Strategies and Survival Trends for Anorectal Melanoma: Is it Time for a Change?. World J Surg.

